# One-year follow-up of functional impairment in inpatients with mood and anxiety disorders – Potentials of the Mini-ICF-APP

**DOI:** 10.1186/s12888-022-03977-1

**Published:** 2022-05-15

**Authors:** Jaeger Susanne, Uhlmann Carmen, Bichescu-Burian Dana, Flammer Erich, Steinert Tilman, Schmid Petra

**Affiliations:** Department of Psychiatry and Psychotherapy I, Ulm University, ZfP Südwürttemberg, Weingartshofer Str. 2, D-88214 Ravensburg, Germany

**Keywords:** Mini-ICF-APP, Disability, Impairment, Participation, Capacities, Functioning

## Abstract

**Background:**

The aim of the study was to investigate functional impairment and its relationship to illness severity in a sample of patients with a diagnosis of mood or anxiety disorder during inpatient treatment and 1 year after discharge.

**Methods:**

Two hundred thirty-nine inpatients with primary diagnoses of mood or anxiety disorders were assessed at baseline and at follow-up by a range of validated instruments. Mini-ICF-APP was used for the assessment of functional impairment, BDI-II for the assessment of clinical symptoms and remission. Sample characteristics and measures of impairment at baseline and at follow-up were analysed descriptively. Symptomatically remitted and non-remitted patients were compared with regard to capacity limitations.

**Results:**

Initially, the sample showed considerable impairment in many capacities, particularly endurance, spontaneous activities, structuring of tasks, competency and flexibility. After 1 year, all capacities significantly improved. The level of impairment was correlated with employment status and severity of clinical symptoms. About 50% of the patients remitted in clinical symptomatology. Retrospectively, the remitted and the unremitted did not differ in functional impairment at baseline but there were considerable differences at follow-up.

**Conclusions:**

Mini-ICF-APP is a useful instrument to monitor functional status and change in psychiatric samples, complementing the usual focus on symptom reduction.

## Background

People with mental disorders are often affected not only by symptoms but also by impairments in activities of daily living and social participation. The International Classification of Functioning, Disability and Health (ICF) of the World Health Organization [1] is a useful tool for systematically describing a person’s available functional *capacities* in reference to a standard environment, as opposed to recording *performance* in the actual environment. Regardless of the actual realisation of abilities (performance), the ICF provides a comprehensive record on the person’s capacities [2]. However, capacity impairment is not an absolute category but needs to be determined in reference to the requirements of a specific context. For example, communication problems due to social phobia may severely impair the capacity of a person to work as a teacher but not to have a job as a research assistant in a laboratory. In medical treatment settings, there is a vital interest in the assessment not only of symptoms but of functioning in order to get a detailed account of the person’s capacities affected by the disorder and draw conclusions with regards to therapy, needs for assistance and prognosis. To quantify the impairment of capacities according to ICF the “Mini-ICF Rating for Mental Disorders” (Mini-ICF-APP) was developed [2]. This rating instrument offers a set of 13 capacities that may be impaired by mental illness and are supposed to be judged in relation to the requirements of a defined context.

As already mentioned, a specific question concerns the relationship between symptoms and functioning. There is already a structural association between the diagnosis of a mental disorder and functional impairment, however. Making a diagnosis according to a standard diagnostic system like DSM-IV presupposes the presence of distinct symptoms (Criterion A) but also a marked distress or disability by the condition (Criterion B) [3]. Studies have repeatedly confirmed a positive correlation between the severity of symptoms and the degree of functional impairment [4–6]. Recently, Rosburg and colleagues [7] showed that patients with different psychiatric diagnoses also have different levels of functional limitations. In a mixed sample, capacity limitations increased with the severity of the symptoms, especially in patients with depressive disorders. Also the development of functional impairment during symptomatic remission has aroused interest: Collard and colleagues [8] found significant differences in functioning between remitted and non-remitted patients with depression 2 years after first diagnosis. Remarkably, functioning in remitted patients was still reduced compared to healthy controls. These results are consistent with the findings of Iancu et al. [9] who followed up a total of 914 participants with persistent, recurrent and fully remitted depression over a 6-year period. Initially, chronically depressed participants showed the most severe impairment, followed by those with recurrent and remitted depression, and all of them were more impaired than healthy controls. At follow-up, symptom improvement was associated with a reduction in impairment, but none of the diagnosed groups reached the level of the healthy controls, i.e., even in remission some impairments persisted. However, these two follow-up studies used self-reports on performance only (WHO DAS II); in contrast, the Mini-ICF-APP is an external rating of capacities that includes both self-reports and anamnestic data as well as observations during the examination situation.

For more than 10 years now, Mini-ICF-APP has proven to be a practicable, economic, reliable and valid assessment instrument for the assessment of capacity limitations. Nevertheless, literature providing detailed reports of the single items’ results in specific samples other research could refer to is sketchy. Some studies [4, 10] have rather small sample sizes (*N =* 120 and 74). With Rosburg et al. [7], for the first time the items’ results from a larger sample are available (*n =* 946), but information on long-term development of functional limitations is still missing. On the other hand, Egger et al. [5] reported data of a remarkable sample of over 3000 inpatients at admission and discharge, but he did not present the 13 single items’ results. An analysis of the single Mini-ICF-APP items with special attention to the long-term course of impairment and in relation to symptomatic remission seems to be overdue.

The aim of this study was to explore capacity impairment degrees according to the mean values of the 13 Mini-ICF-APP items in a sample of 320 psychiatric inpatients with a diagnosis of mood or anxiety disorders. In particular, we were interested in the functional impairment at admission and 1 year after discharge. Finally, we wanted to investigate possible differences in the different capacity dimensions between the symptomatically remitted and non-remitted patients.

## Methods

The study was part of a comprehensive research project on Treatment Pathways of Patients with Anxiety and Depression (PfAD study) [11–13] that had started in 2012. Data collection ended in 2015. The overall projects’ aim was to explore the long-term development of former inpatients with comparable psychiatric diagnoses up to 1 year after discharge from different treatment settings.

### Sample

For the PfAD study, 320 inpatients with an admitting primary diagnosis of the categories mood (F3) or anxiety disorders (including neurotic, stress-related and somatoform disorders) (F4) according to ICD-10 were assessed. Recruitment took place in four different psychiatric inpatient treatment settings: day clinic, two specialized units for crisis intervention and for the treatment of depression, and a psychosomatic clinic, all situated in Southern Germany. Patients diagnosed with comorbid organic, including symptomatic mental disorders (F0), schizophrenia, schizotypal and delusional disorders (F2) or mental retardation (F7) (ICD-10) were excluded. All consecutively admitted adult patients meeting the inclusion criteria were asked for participation and included in the study after given written informed consent. The recruitment stopped in each setting when a number of 80 participants has been reached. The study was approved by the ethics committee of Ulm University (number 284/11). A detailed account of the methodology of the PfAD study is provided in [13].

### Design

**Fig. 1:**
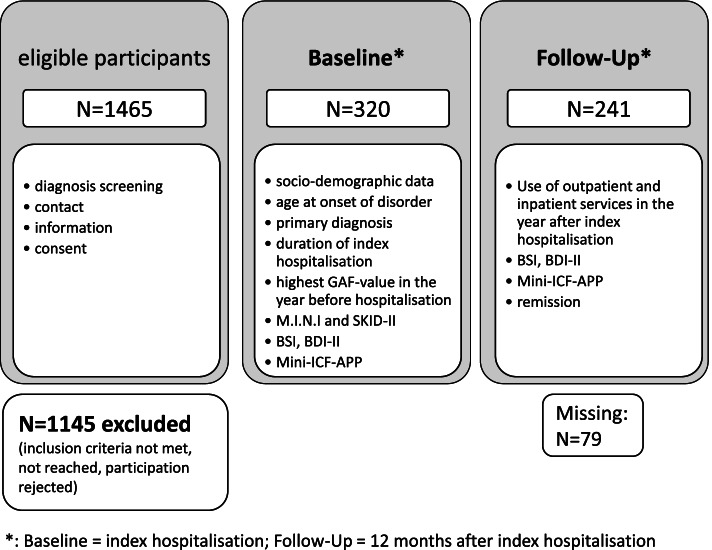
Flow chart

The general framework of the PfAD study was designed to investigate the clinical course and future service use of patients with a diagnosis of mood or anxiety disorder [11]. The prospective longitudinal design of the overall study included four assessments: at the beginning and at the end of the index hospitalisation as well as 6 months and 12 months after discharge. Data gathering included self-assessments and interviews. For the present study’s aim of examining the long-term course of functional impairment in our sample, we decided to use the data assessed during index hospitalisation (baseline) and at 12 months follow-up (follow-up) (Fig. 1).

### Instruments

Socio-demographic, disease-related and treatment-related data were collected using both self- and external assessments. They included age, sex, living in a partnership, less than 9 years of education, supported living, and having a job. We assessed the following clinical characteristics: age at onset of disorder, highest GAF value in the year before admission [3], duration of index hospitalisation, primary diagnosis at discharge, number of co-occurring psychiatric disorders according to the Mini International Neuropsychiatric Interview (M.I.N.I [14].) and the Structured Clinical Interview for DSM-IV, axis II Personality disorders (SCID-II [15]). Additionally, we recorded the use of outpatient and inpatient services in the year after index hospitalisation. In order to assess severity of symptoms we used the global severity index in the Brief Symptom Inventory (GSI; mean value [16]) and Beck Depression Inventory II (BDI-II; sum score [17]).

### Outcome 1: functional impairment

As primary outcome, the level of functional impairment in activities and participation at baseline and at follow-up was assessed using Mini-ICF-APP [2]. This rating instrument consists of 13 items that cover a wide variety of capacities that can be affected by the mental disorder, such as adherence to regulations or flexibility, but also self-care or mobility [2]. Each item is rated on a 5-point scale: 0 = no impairment, 1 = subjective impairment, 2 = observable impairment, 3 = need for assistance, 4 = full impairment, no activity possible in this capacity. A total score can be calculated adding up the ratings of all 13 items. There are versions in German [2, 18], English [19], French [18] and Italian [4, 10]. Several studies have proved its reliability and validity in patients with various mental disorders [4, 10, 19]. The assessments were made by independent study workers not involved in the treatment of the patients. They were trained to rate the items using all available information on the participant (which included a semi-structured interview by the raters) as well as observations during the interview. The rating aimed at assessing the individual’s capacities of general social participation. Accordingly, it was conducted with reference to the individual’s general living context.

### Outcome 2: symptomatic remission

Remission was defined with a cut-off value below 14 points in the BDI-II sum score [13, 17]. BDI-II sum score was chosen as outcome of remission because our assessments showed that patients with primary diagnoses of mood or anxiety disorder did not considerably differ in their baseline BDI-II scores. Regardless of the primary diagnosis, BDI-II sum scores indicated clinically relevant depressive symptoms in the entire sample.

### Statistical analysis

All analyses were performed with SPSS for windows version 27. Initially, we analysed the baseline and follow-up data descriptively, and we tested for a possible attrition bias by comparing study completers (both assessments) with study non-completers. Then we analysed the difference between baseline and follow-up in the various Mini-ICF-APP dimensions (only study completers). Afterwards, we investigated possible correlations between level and quality of impairment and specific sociodemographic and clinical variables. In particular, we were interested in the associations of symptom severity (BDI-II), symptom burden (GSI) and functional impairment (Mini-ICF-APP). We examined them using correlations within and between assessment time points. We compared the mean values of the different capacity limitations in remitted and non-remitted patients. Because of multiple comparisons, we used Bonferroni adjusted *p*-values in all analyses.

## Results

### Sample characteristics

At baseline, a total of 320 inpatients participated in the study. Their mean age was 40.7 years (SD = 12.41), 60.6% were female, 42.2% lived in a partnership, and 67.4% were having a job. About 70% had a primary diagnosis of F3, 30% of F4. On average, inpatient index hospitalisation lasted 48.81 days (SD = 27.23). At admission, the mean BDI-II sum score was 30.07 (SD = 11.59), the mean GSI score was 1.61 (SD = 0.75) (Table 1).

**Table 1:** Characteristics of the total sample and differences between study completers and non-completers at baseline

		Total Sample *n* = 320		Completers *n =* 241		Non-Completers *n =* 79		*p*^1^
**Socio-Demographic Characteristics**								
age	M (SD)	40.74	(12.41)	42.37	(11.88)	35.75	(12.72)	< 0.001^A^
female	n (%)	194	(60.6%)	150	(37.8%)	44	(43.6%)	.423^B^
living in a partnership	n (%)	135	(42.2%)	108	(44.8%)	27	(34.6%)	.116^B^
less than 9 years of education	n (%)	107	(33.4%)	70	(29.0%)	37	(47.4%)	.004^B^
living independently	n (%)	314	(98.1%)	237	(98.3%)	77	(98.7%)	1.00^B^
having a job	n (%)	215	(67.4%)	162	(67.2%)	53	(67.9%)	1.00^B^
age at onset of disorder	M (SD)	28.86	(13.45)	29.44	(13.48)	27.06	(13.29)	.137^A^
highest GAF value in the year before admission	M (SD)	72.14	(14.30)	71.75	(13.52)	73.29	(16.47)	.612^A^
**Clinical Characteristics**								
Primary diagnosis F3	n (%)	225	(70.3)	172	(71.7)	53	(68.8)	.666^B^
Number of comorbid psychiatric disorders	M (SD)	2.43	(1.25)	2.37	(1.20)	2.61	(1.38)	.179^A^
Duration of index hospitalisation (days)	M (SD)	48.81	(27.23)	48.33	(26.24)	50.27	(30.18)	.744^A^
BDI-II total score	M (SD)	30.07	(11.58)	29.66	(11.82)	31.35	(10.75)	.220^C^
GSI score (according to BSI)	M (SD)	1.61	(0.75)	1.59	(0.75)	1.67	(0.73)	.313^C^

^A^ Mann-Whitney-U-test; ^B^ Chi^2^-Test; ^C^ t-test for independent groups; ^1^ *p* <  0.0036 after Bonferroni correction

A total of 241 participants (75%) took part in the follow-up assessment. Attrition bias was examined by comparing the socio-demographic and clinical characteristics of study completers and non-completers. Only mean age differed significantly (U = 6688.00; *p <* 0.001). There was no indication of an attrition bias with regard to clinical severity or other characteristics [13].

### Outcome 1: Mean values of functional impairment at baseline and follow-up

The means of the total score and the 13 capacities at baseline and follow-up are displayed in Table 2. All capacities showed a significant improvement at follow-up. The items indicating the least impairment at both measurement points were self-care, mobility and adherence to regulations. The most impaired capacities at baseline (B) and follow-up (FU) included endurance (M_B_ = 1.98 (SD = 0.96), M_FU_ = 0.94 (SD = 0.96)), spontaneous activity (M_B_ = 1.96 (SD = 1.09), M_FU_ = 0.94 (SD = 1.00)), structuring of tasks (M_B_ = 1.88 (SD = 1.04), M_FU_ = 0.92 (SD = 0.97)), competency (M_B_ = 1.87 (SD = 1.16), M_FU_ = 0.84 (SD = 0.93)), and flexibility (M_B_ = 1.82 (SD = 1.09), M_FU_ = 0.91 (SD = 0.98)), with only minor changes in the ranks of impairment over time. At the same time, these five areas showed the greatest improvement over time. Table 2 also shows the mean changes in the completers in the Mini-ICF-APP total score and in the 13 individual items. It has to be noted that 1 year after discharge, nearly 25% (*n* = 53) of the sample showed no more impairment in any of the single capacities.

**Table 2:** Means, Standard deviation, and rank of values in the mini-ICF-APP total score and the 13 items at baseline and follow-up as well as mean change (Mean and Standard Deviation) at follow-up

	Baseline			Follow-Up			Difference^2^		
	n	M	(SD)	n	M	(SD)	M	(SD)	*p*^1^
ICF total score (mean value)	319	1.56	(0.68)	239	0.78	(0.65)	0.75	(0.76)	< 0.001^A^
adherence to regulations	318	1.18	(1.16)	239	0.41	(0.75)	0.71	(1.25)	< 0.001 ^A^
structuring of tasks	318	1.88	(1.04)	239	0.92	(0.97)	0.93	(1.26)	< 0.001 ^A^
flexibility	318	1.82	(1.09)	238	0.91	(0.98)	0.87	(1.33)	< 0.001 ^A^
competency	315	1.87	(1.16)	234	0.84	(0.93)	1.01	(1.30)	< 0.001 ^A^
judgement	315	1.82	(1.03)	239	0.97	(1.01)	0.80	(1.24)	< 0.001 ^A^
endurance	318	1.98	(0.93)	238	0.94	(0.96)	1.05	(1.13)	< 0.001 ^A^
assertiveness	311	1.62	(1.11)	239	0.85	(0.96)	0.71	(1.29)	< 0.001 ^A^
contact with others	316	1.66	(1.11)	238	0.87	(0.95)	0.79	(1.24)	< 0.001 ^A^
group integration	315	1.36	(1.07)	239	0.84	(1.0)	0.45	(1.27)	< 0.001 ^A^
intimate relations	311	1.51	(1.09)	239	0.84	(0.93)	0.67	(1.24)	< 0.001 ^A^
spontaneous activity	313	1.96	(1.09)	239	0.94	(1.0)	0.99	(1.25)	< 0.001 ^A^
self-care	319	0.78	(0.94)	239	0.34	(0.70)	0.42	(1.04)	< 0.001 ^A^
mobility	316	0.83	(1.07)	238	0.45	(0.80)	0.40	(1.15)	< 0.001 ^A^

^A^ Wilcoxon-Test; ^1^ *p <* 0.0036 after Bonferroni correction; ^2^ difference between Baseline and Follow-Up (only completers): improvements are thus indicated by positive numbers

### Correlations of Mini-ICF-APP and other characteristics

At baseline and at follow-up, there were no significant correlations of Mini-ICF-APP total score and any socio-demographic characteristics, except from employment status. At follow-up, unemployed participants showed a greater impairment score (*r =* − 0.319; *p <* 0.001; *n =* 238). We also examined correlations of Mini-ICF-APP total score at baseline and follow-up and the following clinical characteristics: primary diagnosis F3, total number of co-occurring diagnoses, duration of index hospitalisation, use of outpatient or inpatient services in the year after index hospitalisation. The number of comorbid diagnoses at index treatment correlated with impairment at baseline, but also at follow-up (r_B_ = 0.208, *p <* 0.001, *n* = 319; r_FU_ = 0.294, *p <* 0.001, *n* = 239). Participants who had used outpatient services more intensely (*r =* 0.209; *p <* 0.001; *n* = 228) or who had spent more days in another inpatient treatment after discharge from index hospitalisation (*r =* 0.364; *p <* 0.001; *n* = 229) showed higher Mini-ICF-APP scores at follow-up. Symptom severity and symptom burden were moderately associated with functioning on average (Table 3). GSI and BDI-II scores correlated significantly with Mini-ICF-APP total scores at baseline and follow-up and across assessment time points.

**Table 3:** Correlations of mini-ICF-APP total score at baseline and follow-up and severity of symptoms

	Mini-ICF-APP Total Score at Baseline			Mini-ICF-APP Total Score at Follow-Up		
	n	r	*p*^1^	n	r	*p*^1^
GSI score Baseline	318	0.399	< 0.001^A^	239	0.462	< 0.001^A^
GSI score Follow-Up	206	0.247	< 0.001^A^	205	0.797	< 0.001^A^
BDI-II sum score Baseline	318	0.426	< 0.001^A^	239	0.466	< 0.001^A^
BDI-II sum score Follow-Up	206	0.277	< 0.001^A^	205	0.812	< 0.001^A^

^A^ Pearson correlation; ^1^ *p <* 0.0028 after Bonferroni correction

### Outcome 2: Functional impairment of remitted and non-remitted patients

**Fig. 2:**
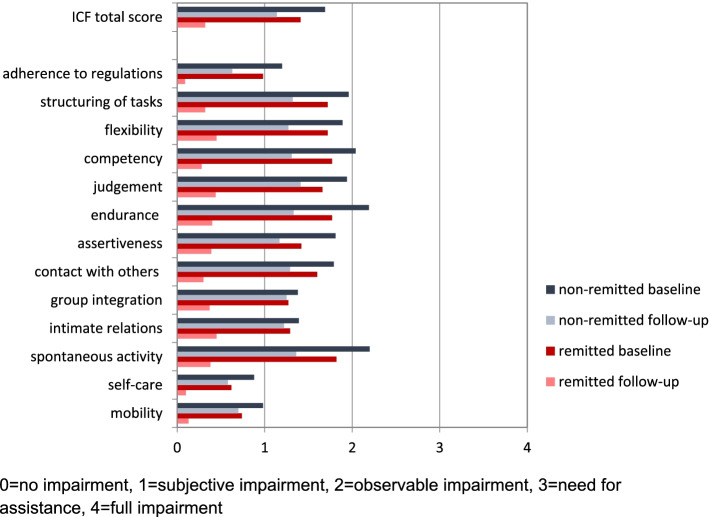
Mean Values of the Mini-ICF-APP Total Score and the 13 Single Items of the Symptomatically Non-Remitted and Remitted Patients at Baseline and Follow-Up. 0 = no impairment, 1 = subjective impairment, 2 = observable impairment, 3 = need for assistance, 4 = full impairment

At follow-up, 94 (45.4%) participants met the criteria for remission according to BDI-II. Figure 2 shows that functional impairment of both remitted and non-remitted patients decreased over time. However, at baseline, the level of impairment of the later remitted and non-remitted patients was comparable. There were no significant differences except for endurance (U = 3985.00; *p <* 0.001). At follow-up, there were significant differences between remitted and non-remitted patients in all items (Table 4). The most heavily impaired capacities in non-remitted patients were judgement, spontaneous activity, and endurance. The main remaining limitations in remitted patients concerned judgement, flexibility and intimate relationships. However, means below 1 in Mini-ICF-APP always indicate the absence of or only small problems without the need of external assistance. The areas of self-care, adherence to regulations, and mobility played a minor role in both groups.

**Table 4:** Mini-ICF-APP scores at follow-up in remitted and non-remitted patients

	Non-Remitted^1^			Remitted^1^			*p*^2^
	n	M	(SD)	n	M	(SD)	
total score (mean value)	111	1.14	(0.63)	94	0.32	(0.33)	< 0.001^A^
adherence to regulations	111	0.63	(0.87)	94	0.09	(0.32)	< 0.001^A^
structuring of tasks	111	1.32	(1.01)	94	0.32	(0.55)	< 0.001^A^
flexibility	110	1.27	(1.05)	94	0.45	(0.70)	< 0.001^A^
competency	108	1.31	(0.93)	93	0.28	(0.54)	< 0.001^A^
judgement	111	1.41	(1.02)	94	0.44	(0.68)	< 0.001^A^
endurance	110	1.33	(0.95)	94	0.40	(0.68)	< 0.001^A^
assertiveness	111	1.17	(1.04)	94	0.39	(0.59)	< 0.001^A^
contact with others	110	1.29	(0.96)	94	0.30	(0.55)	< 0.001^A^
group integration	111	1.25	(1.07)	94	0.37	(0.69)	< 0.001^A^
intimate relations	111	1.22	(0.98)	94	0.45	(0.73)	< 0.001^A^
spontaneous activity	111	1.36	(0.95)	94	0.38	(0.64)	< 0.001^A^
self-care	111	0.58	(0.86)	94	0.10	(0.42)	< 0.001^A^
mobility	110	0.70	(0.93)	94	0.13	(0.42)	< 0.001^A^

^A^ Mann-Whitney U test; ^1^ Categorization based on BDI-II values; ^2^ *p <* 0.0036 after Bonferroni correction

## Discussion

Mini-ICF-APP is frequently used to assess therapeutic progress in psychiatric rehabilitation and is often applied for the assessment and prediction of a return to work. As initially stated, there are several studies on clinical samples with different psychiatric diagnoses, but often only values at hospital admission and discharge are reported. This study provides a more detailed picture of the long-term progress of formerly hospitalised patients. We examined a large and – in contrast with other studies [4, 5, 19, 20] – narrowly defined diagnostic sample of patients with mood and anxiety disorders as primary diagnoses. Participants were recruited in diverse inpatient treatment settings available in Germany. Initial severity of illness ranged from acute suicidality to full job functioning until admission. Furthermore, our follow-up 1 year after discharge from index hospitalisation adds valuable evidence to the results of former studies [5, 19].

During hospitalisation, our sample was heavily burdened by limited capacities. This is especially true for structuring of tasks, endurance, spontaneous activities, flexibility, and competency, which may be closely related to typical symptoms of acute mood and anxiety disorders. The levels of impairment were comparable to those found in outpatients with schizophrenia [10] and the participants of a multidisciplinary work disability evaluation [7], but were distinctly higher than in psychosomatic rehabilitation inpatients [21]. However, caution is advised to compare these results directly because a similar reference context for judgement of Mini-ICF-APP is necessary in order to draw valid conclusions. In some of these studies, the context is not explicitly specified; others focus primarily on working abilities [21].

However, each of these limited capacities improved after 1 year, especially those most affected. This confirms that the Mini-ICF-APP is able to measure changes and can be used to monitor therapeutic progress. The distribution of mean scores 1 year after discharge is strongly skewed, indicating high rates of complete recovery, also in terms of functional impairment. Nevertheless, a smaller fraction still needed external support in some areas due to limited abilities. The extent of improvement varied for the individual items. Therefore, it seems useful to take a close look at the individual items, i.e. 13 capacities each for itself [4, 10]. Similar to other studies [20, 22], our results indicate a strong relationship between the capacities assessed by Mini-ICF-APP and working abilities. This underlines the external validity of the instrument as a comprehensive assessment of functional capacity.

On average, there was a moderate correlation between the severity of symptoms and limitations of functioning, and symptomatic improvement appeared to be associated with functional improvement. This suggests that symptom severity varies with capacity impairment, but that, at the same time, symptoms and capacity impairments are distinct aspects of illness. At follow-up, the prevalence of symptoms was highly correlated with the level of functioning. This is a strong argument for increased therapeutic efforts to decrease the symptom severity, because at the same time this may increase functioning.

At baseline, there were no significant differences in the impairment of capacities between the later non-remitted and remitted patients, except for endurance. Both groups started at a high level of impairment and improved over time, but the symptomatically remitted patients improved considerably more. There is a fair bit of literature on persisting functional impairment even after symptomatic remission in depression [8, 9]. Some capacities recover only with a time lag after complete symptom remission, which might impede the return to everyday life. Even the mean impairment scores of our remitted subsample were not zero at the end. Thus, several remitted patients still had minor problems even when they did not need help. This is consistent with Collard and colleagues [8], who demonstrated persistent differences between remitted depressed patients and healthy controls.

Regarding the limitations of our study, our sample was limited to inpatients with mood and anxiety disorders. On the one hand, this was an opportunity to take a more detailed look at the group of inpatients with the most prevalent mental disorders (apart from substance-related disorders) in Germany [23]. On the other hand, these diagnostic categories include a heterogeneous spectrum that can range from depression to manic episodes or from specific phobias to reactions on severe stress. Although depressive episodes and anxiety were the most common diagnoses in our sample, there was still a percentage of participants with other symptoms. Thus, we may not attribute our results solely to depression and anxiety disorders but keep in mind the complete spectrum of F3 and F4 disorders according to ICD-10. Moreover, we refrain from generalizing our results to the great number of patients who suffer from anxiety or depression but have never taken advantage of inpatient treatment before. The use of BDI-II as a measure of symptomatic outcome might be criticised as it is intended to assess symptoms of depression. Self-assessment symptom scales, however, cannot be specific for recording a particular disorder. The symptoms asked about in the scales are nonspecific and may be present in many disorders (e.g., sleep, concentration, or various emotional disturbances). Self-assessment scales are therefore primarily useful for expressing patients’ self-reported levels of burden, but do not assess “diagnoses” in a strict sense. Indeed, there was considerable overlap between comorbid diagnoses in our sample [11], and the problems recorded with BDI-II were equally relevant to patients with a primary diagnosis of mood or anxiety disorders. The sample’s attrition rate during the study was considerable despite the lack of a systematic bias beyond the age variable. We cannot rule out an attrition bias in the completers due to unassessed factors. Finally, as we did not assess the functional impairment at the moment of discharge, we cannot draw any conclusion on the progress of functional recovery and the treatment effect during hospitalisation.

## Conclusion

Most studies have used the Mini-ICF-APP in the context of rehabilitation and sick leave [2, 19, 20, 22]. Our study provides results and reference values of a clinical population with mood or anxiety disorders, from different inpatient treatment settings, and with different degrees of severity [11]. We have found this tool to be useful in the context of general psychiatry as well. It can be used to precisely determine capacity limitations and to support treatment planning beyond the focus on symptom reduction. We provided follow-up data over a one-year interval, after the former inpatients had returned to their “normal” life. Our results show that the Mini-ICF-APP is a valuable instrument to monitor functional status and change in psychiatric samples, both in terms of the total score and the individual items. Our reference values of symptomatically remitted and non-remitted subgroups can hopefully contribute to the future standardization of the Mini-ICF-APP, and thus also to promoting the application of the Mini-ICF-APP in clinical practice.

## Data Availability

The datasets used and analysed during the current study are available from the corresponding author on reasonable request.

## Abbreviation

Mini-ICF-APP: Mini-ICF Rating for Mental Disorders
GAF: Global Assessment of Functioning
M.I.N.I.: Mini International Neuropsychiatric Interview
BDI-II: Beck Depression Inventory II
GSI: Global severity index in the Brief Symptom Inventory
