# Correction: One-year follow-up of functional impairment in inpatients with mood and anxiety disorders – Potentials of the Mini-ICF-APP

**DOI:** 10.1186/s12888-022-04051-6

**Published:** 2022-06-22

**Authors:** Susanne Jaeger, Carmen Uhlmann, Dana Bichescu-Burian, Erich Flammer, Tilman Steinert, Petra Schmid

**Affiliations:** Department of Psychiatry and Psychotherapy I, Ulm University, ZfP Südwürttemberg, Weingartshofer Str. 2, D-88214 Ravensburg, Germany

**Correction: BMC Psychiatry 22, 334 (2022)**

**https://doi.org/10.1186/s12888-022-03977-1**

Following the publication of the original article [1], the authors identified errors in the authors names. The given names and family names were erroneously transposed.

The incorrect authors names are:

Jaeger Susanne*, Uhlmann Carmen, Bichescu-Burian Dana, Flammer Erich, Steinert Tilman and Schmid Petra

The correct authors names are:

Susanne Jaeger*, Carmen Uhlmann, Dana Bichescu-Burian, Erich Flammer, Tilman Steinert and Petra Schmid

The author group has been updated above and the original article [1] has been corrected.
